# Antitumor activity of cobrotoxin in human lung adenocarcinoma A549 cells and following transplantation in nude mice

**DOI:** 10.3892/ol.2014.2467

**Published:** 2014-08-21

**Authors:** JIAN SHEN, YAN XIE, MEI-LIN SUN, RONG HAN, ZHENG-HONG QIN, JING-KANG HE

**Affiliations:** 1 Department of Cardiothoracic Surgery, The First Affiliated Hospital of Soochow University, Suzhou, Jiangsu 215006, P.R. China; 2 Department of Pharmacology and Laboratory of Aging and Nervous Diseases, School of Pharmaceutical Science, Soochow University, Suzhou, Jiangsu 215123, P.R. China

**Keywords:** cobrotoxin, human lung A549 cells, autophagy, p38, mitogen-activated protein kinase

## Abstract

The aim of the present study was to investigate cobra neurotoxin (cobrotoxin) activity in A549 cell lines transplanted into nude mice, and to explore its molecular mechanism. The 3-(4,5-dimethylthiazol-2-yl)-2,5-diphenyltetrazolium bromide (MTT) method was used to detect the growth inhibition rate of cobrotoxin in human lung A549 adenocarcinoma cells and HFL1 lung fibroblasts. Cell colony formation assays were performed to determine the effect of cobrotoxin on A549 cell colony formation, and transmission electron microscopy was used to detect cobrotoxin autophagy. In addition, western blot analysis was performed to determine the effect of 3-methyl adenine (3-MA) activity on the inhibition of autophagy, SB203580 inhibition of the p38-mitogen-activated protein kinase (MAPK) pathway, and Beclin 1, LC3, p62, p38 and phosphorylated (p)-p38 protein expression. Nude mice were injected with human lung A549 cells, and intervention and control groups were compared with regard to tumor suppression. The MTT assay revealed that various concentrations of cobrotoxin inhibited growth of A549 cells, but not HFL1 cells. A549 cell colony formation decreased and autophagosome activity was significantly increased compared with the controls. Following 3-MA administration, SB203580 autophagosome activity decreased, and following cobrotoxin administration, Beclin 1, p-p38, and LC3-II protein expression significantly increased, whereas p62 expression significantly decreased. Following 3-MA inhibition of autophagy, Beclin 1, LC3-II and p62 expression increased. Furthermore, following SB203580 inhibition of the p38-MAPK pathway, Beclin 1, p-p38, LC3-II and p62 protein expression increased. Cobrotoxin exhibited inhibitory activity on the human lung cancer A549 cells transplanted into the nude mice, suppressing the tumor growth rate by 43.4% (cobrotoxin 40 μg/kg group). However, following the addition of 3-MA (10 mmol/kg) and SB203580 (5 mg/kg), the suppression of the tumor growth rate decreased significantly. Cobrotoxin inhibits the growth of human lung cancer A549 cells *in vitro* and A549 cells transplanted into nude mice. Furthermore, the induction of autophagy may be associated with the activation of the p38-MAPK pathway.

## Introduction

At present, non-small cell lung cancer (NSCLC) is one of the most common types of malignant tumor. The US Centers for Disease Control and Prevention have shown that the number of mortalities from lung cancer is higher than any other type of cancer (1). Lung adenocarcinoma accounts for 40% of all lung cancers, the incidence is predominant in the female popluation and is not generally attributed to cigarette smoking. Lung adenocarcinoma has an five-year survival rate of 11.6% in the USA (2) due to the fact that numerous patients are not diagnosed early and therefore lose the opportunity to undergo complete surgical resectioning. Treatment usually involves surgery, however, this is often inadequate. Studies regarding NSCLC have been exhaustive and research has progressed, particularly in the search for natural anti-tumor treatments (3). Cobra neurotoxin (cobrotoxin), derived from cobra venom, mainly consisting of a postsynaptic nerve toxin. Cobrotoxin is capable of combining with acetylcholine receptors, which may be the underlying mechanism for its anti-inflammatory, immune adjustment and pain easing qualities, additionally it has been shown to exhibit antineoplastic activity (4,5). In the present study, the effect of cobrotoxin on human lung cancer A549 cells was investigated to determine a potential molecular mechanism, in order to provide a theoretical basis for its application in clinical use.

## Materials and methods

### Cell culture

Human A549 lung glandular cancer cells and human lung fibroblast cells that demonstrated adherent growth were obtained from the Shanghai Institute of Cell Biology (Shanghai, China). The A549 cells were maintained in RPMI-1640 culture medium containing 10% fetal bovine serum. Human lung fibroblasts were maintained in Dulbecco’s modified Eagle’s medium containing 10% calf serum. The two cell lines were incubated at 37°C in a humidified atmosphere of 5% CO_2_, and were harvested when they reached 80–90% fusion with 0.25% trypsin digestion. Cells in the logarithmic phase were used for this study. The study was approved by the ethics committee of Soochow University (Suzhou, China).

### Drugs, reagents and equipment

Cobrotoxin was a gift from the Department of Pharmacy at the Suzhou University Medical College (Suzhou, Jiangsu, China), while the 3-methyl adenine (3-MA), dimethyl sulfoxide (DMSO) and 3-(4,5-dimethylthiazol-2-yl)-2,5-diphenyltetrazolium bromide (MTT) were purchased from Sigma-Aldrich (St. Louis, MO, USA). SB203580 was purchased from Beyotime Institute of Biotechnology (Haimen, China) and an automatic enzyme standard instrument (Benchmark) was purchased from Bio-Rad (Hercules, CA, USA). A Heracell 150 carbon dioxide incubation box was purchased from Thermo Fisher Scientific (Rockford, IL, USA). SDS-PAGE apparatus (Mini-PROTEAN Tetra Electrophoresis System and Mini-PROTEAN 3 Dodeca Cell, Bio-Rad) and a FEI TECNAI 10 transmission electron microscope (TEM; Philips, Amsterdam, Holland) were also utilized.

### Cell growth inhibition (MTT assay)

A single cell suspension (1×10^5^ cells/ml) of HFL1 and A549 cells in the logarithmic phase was prepared following 0.25% trypsin digestion and seeded on a 96-well culture plate (100 μl/well). Adherent cells were observed subsequent to 24 h. Cobrotoxin concentrations of 5, 10 and 20 μg/ml were added to 6 wells in each row, leaving one negative control group and one set of blank wells. After 48 h, an MTT (5 mg/ml) assay was performed and the cells were incubated for 4 h. Next, DMSO (150 μl) was added to each well and the plates were agitated gently for 10 min. Optical density (OD) was then measured at a wavelength of 570 nm. Assays were performed in triplicate and the mean values were calculated. The cell proliferation inhibition rate (%) was calculated as follows: Cell proliferation inhibition rate (%) = (1 - mean OD value / control mean OD value) × 100.

### Cell colony tablet cloning

The A549 cells were harvested at the logarithmic phase following 0.25% trypsin digestion into a single cell suspension (400 cells/ml), and placed into 24 wells (200 cells/well). The cells were then incubated for 24 h, following which, 5, 10, and 20 μg/ml cobrotoxin was added, and a negative control group was created. After 48 h, RPMI-1640 medium was replaced (once every 2–3 days). After two weeks, the cells were fixed with methanol, stained with Giemsa and observed microscopically for a cell count of >50 cell colonies. The colony formation inhibition rate (%) was calculated as follows: Colony formation inhibition rate (%) = (control colony formation rate - experimental colony formation rate / control colony formation rate) × 100.

### TEM

The A549 cells in the logarithmic phase were harvested, digested with 0.25% and prepared in a single cell suspension, whereby cell density was adjusted to 1.0×10^5^ cells/ml and the cells were plated. RPMI-1640 culture medium containing 10% fetal bovine serum was added and the cells were left overnight. Culture medium was added to create the following groups: 10 μg/ml cobrotoxin, 10 μg/ml cobrotoxin + 5 mmol/ml 3-MA and 10 μg/ml cobrotoxin + 10 μmol/ml SB203580. The negative control contained only RPMI-1640 medium. After 48 h in the nutrient solution 2–3 drops of 2.5% glutaraldehyde were added, followed by centrifugation at 3,000 rpm, at a centrifugal radius of 16 cm. Following separation of the supernatant, 2.5% glutaraldehyde was added, microwaved for 5–10 sec and fixed for 1 h at 4°C. Using a hook anatomical needle, the cell mass was removed and cut into 1-mm^3^ blocks.

The generation of tissue blocks was performed by fixing samples with 2.5% glutaraldehyde and refrigerating at 4°C for >2 h, followed by three washes with 0.1 mol/l phosphate buffer for 15 min each and fixation with 1% osmic acid for 2 h. This was followed by a second rinse with 0.1 mol/l phosphate buffer three times for 15 min each, dehydration for 15 min and three washes with 50% ethanol, followed by drying with 3% acetic acid (70% ethanol uranium) overnight. Next, gradient dehydration (90% ethanol:90% acetone, 1:1) was performed for 15 min each, followed by 100% acetone ketal dehydration at room temperature (15 minutes each) three times, and embedding (pure acetone:embedding liquid, 1:1) at room temperature overnight (pure liquid embedding at 37°C for 3 h). Finally, the blocks were cured at 45°C for 6 h, then at 60°C for 24 h. Using an LKB-1 (Pharmacia, Stockholm, Sweden) type ultra-thin slicing machine, each sample was then cut at a thickness of 50–60 nm, stained with a citrate lead piece and visualized. Images were captured using TEM.

### Western blotting determination of Beclin 1, LC3, p62, p38 and phosphorylated (p)-p38 protein expression

The A549 cells were harvested in the logarithmic phase, digested with 0.25% trypsin, prepared in a single cell suspension, with the cell density adjusted to 1.0×10^5^ cells/ml, and plated. Cobrotoxin was added in the following concentrations: 5, 10 or 20 μg/ml, 10 μg/ml cobrotoxin + 5 mmol/ml 3-MA and 10 μg/ml cobrotoxin + 10 μmol/ml SB203580 culture. A negative control containing only RPMI-1640 medium was created. The mixture was transferred to an Eppendorf tube (Eppendorf, Hamburg, Germany) on ice and subjected to ultrasonic irradiation (JY96-II, Haishu Kesheng Ultrasonic Equipment Co., Ltd., Ningbo, China) for 30 min, followed by centrifugation at 12,000 rpm, at a centrifugal radius of 16 cm, for 5 min at 4°C. The protein concentration in the supernatant was determined using the BCA method (Pierce, Rockford, IL, USA). The samples were boiled, and the proteins were separated using SDS-PAGE. Blots were subsequently transferred to nitrocellulose membranes (Bio-Rad) and hybridized. The primary antibodies used were rabbit anti-human Beclin-1 (1:500; BioVision, Inc., Milpitas, CA, USA), LC3 (1:500; BioVision, Inc.), p38 (1:1,000; Cell Signaling Technology, Inc., Beverly, MA, USA) and p-p38 (1:1,000; Cell Signaling Technology, Inc.) monoclonal antibodies, with β-actin used as an internal control. Secondary antibodies were used at a 1:5,000 diultion and labeled with horseradish peroxidase in blocking solution for 1 h at room temperature. A chemiluminescence assay was performed to determine protein expression. Optical densities of respective protein bands were analyzed using Sigma Scan Pro 5 (Sigma-Aldrich) and normalized with loading control.

### Nude mice transplantation tumor model

Following disinfection of the dorsal skin, 28 female nude mice (age, 35 days; weight, 18–24 g) were injected with A549 cells in the logarithmic phase, with a cell density adjusted to l.0×10^8^ cell/ml, from a cell suspension of 100 μl (total of 1×10^7^ cells). Following injection, the animals were closely observed each day for their activity levels, mental state, food intake and defecation amounts. Vernier calipers were used to measure the size of subcutaneous transplantation tumors and the animals were weighed twice a week.

### Calculation of tumor inhibition for each intervention group

Nine days after inoculation, tumor nodules grew to 4–6 mm in size. Each group was randomly divided into four groups of seven mice, and received 40 μg/kg cobrotoxin, 40 μg/kg cobrotoxin + 10 mmol/kg 3-MA, 40 μg/kg cobrotoxin + 5 mg/kg SB203580 or 0.9% NaCl (negative control group). During the same week as cell transplantation, the experimental animals received cobrotoxin doses subcutaneously, as described, while control animals received saline injections. The mice were sacrificed four weeks after treatment. The nodules were excised and weighed, and tumor inhibition rates were calculated as follows: Tumor inhibition rate (%) = (1 - weight of experimental group tumor / weight of control group tumor) × 100.

### Statistical analysis

Data are presented as the mean ± standard deviation. Experimental and control groups were compared using the two sample t-test. Comparison between the groups was performed using single factor analysis of variance. P<0.05 was considered to indicate a statistically significant difference. SPSS version 15.0 (SPSS, Inc., Chicago, IL, USA) was used for statistical analysis.

## Results

### Cobrotoxin inhibition of A549 and HFL1 cell growth

After 48 h, significant inhibition of the A549 cells was exhibited, as determined by the MTT method (P<0.05), at rates of 24.1, 32.3 and 56.4%, following treatment with 5, 10 and 20μg/ml cobrotoxin, respectively (Table I). Cobrotoxin did not appear to affect HFL1 cell growth.

### Cobrotoxin impact on A549 cell colony formation

Compared with the controls, cobrotoxin significantly inhibited A549 cell colony formation at 48 h in a dose-dependent manner (Table II).

### TEM analysis regarding A549 cell morphology and autophagy

**Figure 1:**
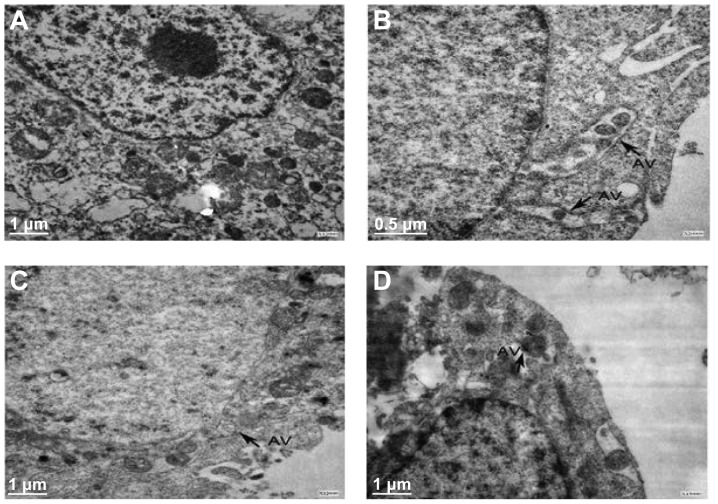
Transmission electron microscopy was used to observe ultrastructural changes in A549 cells compared with plasma controls. (A) Control, (B) cobrotoxin, (C) cobrotoxin + 3-MA and (D) cobrotoxin + SB203580. Cobrotoxin, cobra neurotoxin; 3-MA, 3-methyl adenine.

TEM was used to observe structural changes in the A549 cells compared with the plasma controls. Following 48 h of cobrotoxin treatment, a large number of cytoplasmic double-membrane structures were observed, with gradual extension and bending, containing cytoplasm components and lysosome organelles, with signs of autophagy and glycogen accumulation in the cells. In the cobrotoxin + 3-MA and cobrotoxin + SB203580 groups, autophagy was significantly reduced (Fig. 1).

### Western blot analysis of p62, Beclin 1, LC3, p-p38 and p38 protein expression levels

**Figure 2:**
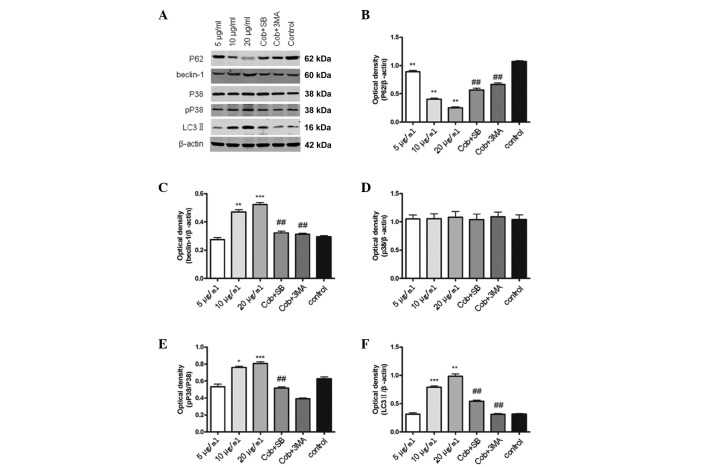
Effects of cobrotoxin on p62, Beclin-1, p38, p-p38 and LC3-II protein expression in A549 cells. Protein levels were analyzed with immunoblotting. Optical densities of respective protein bands were analyzed using Sigma Scan Pro 5 and normalized with loading control (β-actin) vs. control group. ^*^P<0.05, ^**^P<0.01 and ^***^P<0.001 vs cobrotoxin group (10 μg/ml). ^#^P<0.05 and ^##^P<0.01 vs. the 10 μg/ml cobrotoxin group. p-, phosphorylated; cobrotoxin, cobra neurotoxin; 3-MA, 3-methyl adenine.

Compared with the controls, Beclin 1, p-p38 and LC3-II protein expression in the cobrotoxin treated group was significantly increased, and p62 protein expression decreased significantly, in a dose dependent manner. Compared with the cobrotoxin group (10 μg/ml), in the cobrotoxin + SB203580 intervention group, Beclin 1, p-p38 and LC3-II protein expression decreased, and p62 protein expression was increased significantly, however, no significant differences were identified in p38 protein expression. Compared with cobrotoxin group (10 μg/ml), in the cobrotoxin + 3-MA intervention group, Beclin 1 and LC3-II protein expression were decreased significantly, whereas p62 was increased significantly (Fig. 2).

### In vivo tumor suppression of human lung cancer A549 cells transplanted in nude mice

A total of 28 Balb/c nude mice were subcutaneously injected with the A549 cells. After 9 days, the mice grew subcutaneous round or oval tumor nodules with clear palpable boundaries, and demonstrated skin aging. There was no evident change in weight, however, activity declined with tumor growth. There was no significant difference identified between the groups with regard to body morphology or tumor size at 12–16 days post-transplantation. At 20 days post-cobrotoxin administration, tumor growth slowed, the animals were lively, no significant change in weight was identified and there were no deaths. After 30 days, the growth of the tumor nodules was more rapid, and the animals lost weight and appeared lifeless with decreased activity, although without any deaths. In the cobrotoxin + 3-MA and cobrotoxin + SB203580 subcutaneous transplantation groups, the tumor nodules of the animals grew slightly faster. The animals were sacrificed and the subcutaneous tumor nodules were removed; no distant metastases were observed. The tumor inhibition rate of the tumors transplanted into the nude mice were 43.4, 25.0 and 31.5%, for the cobrotoxin, cobrotoxin + 3-MA and cobrotoxin + SB203580 groups, respectively (P<0.01 compared with the control group; Table III).

The intervention and control groups differed significantly with regard to tumor weight (P<0.01 for the cobrotoxin + 3-MA intervention group and P<0.05 for the cobrotoxin + SB203580 intervention group, compared with the cobrotoxin group).

## Discussion

Cobrotoxin exhibits an analgesic effect and has been approved by the Food and Drug Administration for clinical application. Recently, cobrotoxin has been reported to exhibit an antitumor effect, however, the specific mechanism remains unclear. It has been speculated that its antineoplastic effect may be associated with the N-type acetylcholine receptor (6). In the present study, cobrotoxin was found to exhibit an evident anti-tumor effect in the A549 cells (inhibition rates of 24.1% at 5 μg/ml, 32.3% at 10 μg/ml and 56.4% at 20 μg/ml). In addition, cobrotoxin inhibited A549 cell colony formation (27.1% at 5 μg/ml, 43.9% at 10 μg/ml and 54.8% at 20 μg/ml). Furthermore, TEM revealed the separation of autophagy and cytoplasm components. 3-MA and SB203580 autophagy following intervention was reduced, demonstrating that cobrotoxin antitumor effects, the induction of autophagy and the activated p38-mitogen-activated protein kinase (MAPK) pathways are closely associated.

Autophagy is a lysosomal function of the cell degradation process, however, the mechanism of autophagy is unclear (7). In autophagy, LC3-I is converted to LC3-II and results in a novel autophagy membrane, therefore, LC3, particularly type II, is usually used to indicate autophagy in mammalian cell proteins (8,9). Autophagy genes, such as Beclin 1, are specific to autophagy in mammals and also reflect its occurrence (10). The present study observed that following treatment of the A549 cells with cobrotoxin, Beclin 1 and LC3-II protein expression was increased, and p62 protein-induced autophagy was also confirmed. 3-MA inhibited autophagy and increased p62, Beclin 1 and LC3-II protein expression, further confirming autophagy.

Autophagy exhibits a dual role in tumor development; in the case of an insufficient blood supply autophagic tumor cells receive energy metabolism, which maintains their growth and protects the cells. However, autophagic cell death may also occur (11,12). The MAPK signal transduction pathway is an important signaling system that stimulates signal transduction of extracellular stimuli to cells, mediating proliferation, differentiation, transformation, apoptosis and autophagy (13–16). The present study observed that p38 and p62 protein expression increased following MAPK pathway-specific SB203580 inhibition, whereas Beclin 1 and LC3-II protein expression decreased, and thus, we hypothesize that cobrotoxin-induced autophagy of A549 cells may activate the p38-MAPK signaling pathway.

Nude mice were injected with the A549 lung cancer cells and tumor growth inhibition was observed following cobrotoxin treatment, without evident adverse effects on the mice and with no significant differences identified between the treatment and control groups. The cobrotoxin + 3-MA and cobrotoxin + SB203580 intervention groups showed rapid tumor growth compared with the cobrotoxin group, a further sign of cobrotoxin involvement in the p38-MAPK pathway for A549 cell autophagy. Cobrotoxin inhibited A549 cell growth *in vitro*, and this effect may be due to the activation of the p38-MAPK pathway autophagy process. The present study aids in the understanding of the mechanism of cobrotoxin-induced autophagy in A549 cells, and provides new insight into antitumor combinations for lung cancer therapy.

## Figures and Tables

**Table I:** Effect of cobrotoxin on the growth of A549 cells.

Cobrotoxin concentration, μg/ml	Optical density (mean ± SD)	P-value	Inhibition rate, %
Control	0.741±0.121		
5	0.562±0.127	0.017	24.1
10	0.501±0.034	0.004	32.3
20	0.323±0.018	0.001	56.4

SD, standard deviation; cobrotoxin, cobra neurotoxin.

**Table II:** Effect of cobrotoxin on colony formation.

Cobrotoxin concentration, μg/ml	Colony number (mean ± SD)	P-value	Inhibition rate, %
Control	136.4±15.1		
5	99.3±6.5	0.027	27.1
10	76.5±11.7	0.013	43.9
20	61.5±12.1	0.005	54.8

SD, standard deviation; cobrotoxin, cobra neurotoxin.

**Table III:** Effects of each intervention group on tumor weight and tumor inhibition in a nude mouse subcutaneous transplantable tumor model.

Group	n	Average tumor weight, g (mean ± SD)	Tumor inhibition rate, %
Control	7	0.76±0.13	
Cobrotoxin	7	0.43±0.24	43.4
Cobrotoxin + 3-MA	7	0.57±0.15	25.0
Cobrotoxin + SB203580	7	0.52±0.07	31.5

SD, standard deviation; cobrotoxin, cobra neurotoxin; 3-MA, 3-methyl adenine.
